# Hyperglycemia Induces Neutrophil Extracellular Traps Formation Through an NADPH Oxidase-Dependent Pathway in Diabetic Retinopathy

**DOI:** 10.3389/fimmu.2018.03076

**Published:** 2019-01-08

**Authors:** Luoziyi Wang, Xin Zhou, Yizhou Yin, Yuxin Mai, Desai Wang, Xuedong Zhang

**Affiliations:** ^1^Department of Ophthalmology, The First Affiliated Hospital of Chongqing Medical University, Chongqing, China; ^2^Chongqing Key Laboratory of Ophthalmology, Chongqing Eye Institute, Chongqing, China; ^3^Laboratory of Stem Cell Biology, State Key Laboratory of Biotherapy, West China Hospital, Sichuan University, Chengdu, China

**Keywords:** type 2 diabetes mellitus, diabetic retinopathy (DR), hyperglycemia, neutrophil extracellular traps (NETs), NADPH oxidase

## Abstract

Neutrophil extracellular traps (NETs), the product of NETosis, is found to localize pathogens and crystals in immune response. Recent studies have found that excessive NETs lead to disease conditions such as diabetes and its complications like diabetic retinopathy (DR). However, the correlation between NETs and high glucose or DR remains unclear. Here, we found NETs level was significantly increased in the serum of diabetic patients, especially in proliferation diabetic retinopathy (PDR) patients. High glucose dramatically increased NETs production in diabetic individuals with time prolonging. The activation of NADPH oxidase was involved in the NETs process which is triggered by high glucose. Moreover, we verified the infiltration of neutrophils in the eyes and adhesion to vascular endothelial cells in diabetic rat models. NETs formation was observed in the vitreous bodies and retinas of diabetic individuals, which indicates NETs may play a role in the pathogenesis of diabetic retinopathy. Furthermore, anti-VEGF therapy downregulates NETs production indicating that NADPH oxidase-derived ROS may be another signaling pathway involved in anti-VEGF therapy.

## Introduction

Diabetes mellitus (DM) is one of the most common metabolic disorders in both developing and developed countries (1, 2). The epidemiological data showed that it is affecting 1 in 10 people all over the world. It was estimated by the World Health Organization (WHO) that the number of people living with this disease will reach 366 million by 2030 (3). In the meantime, the International Diabetes Federation (IDF) reported that the number will reach 693 million by 2045 (4, 5). Persistently high circulating sugar/glucose in the blood can influence cellular functions and attack macro- and microvessels throughout the whole organs (3). IDF announced that there are almost 5 million people dying from diabetes-specific complications such as retinopathy and nephropathy than high-risk infectious diseases such as AIDS and tuberculosis (6).

Diabetic retinopathy (DR) is becoming the leading cause of preventable partial or total vision impairment in working-age and elderly people in most parts of the world (7, 8). There are plenty of clinical researches proving that chronic imbalanced sugar/glucose in the blood do damage to retina microvasculature, make blood-retinal barrier (BRB) broken down, fluid leaked, and intra-retinal hemorrhage in early non-proliferative phase of DR (NPDR), and cause retinal neovascularization in proliferative phase of DR (PDR) (9–11). Besides, researchers found that chronic low-grade inflammation is intimately correlated with the pathogenesis of DR which is mainly caused by the activation of the innate immune system (12, 13). As the most abundant type of leukocytes (up to 65%), neutrophils derive from bone marrow and are released into peripheral circulation (14, 15). They function actively in innate immunity with an ability for the first line of cellular defense. When diabetes progress to the stage of ocular complications, long-term hyperglycemia destroys the integrity of BRB. Under that circumstance, neutrophils pass through the uncompleted BRB, infiltrate into retinas and choroids, and adhere to vascular endothelial cells. Moreover, increasing neutrophils infiltration in the retinas and choroids of DR patients accelerates neutrophil-endothelial cell adhesion as well as retinal leukostasis which contributes to disease progression (16, 17).

NETosis, a novel neutrophil-specific cell death process, which differs from necrosis and apoptosis, is characterized by releasing neutrophil extracellular traps (NETs) to the extracellular space to defend pathogens and some crystals (18–21). It consists of decondensed chromatin structural framework decorated with 20 kinds of granular antimicrobial proteins such as myeloperoxidase (MPO), neutrophil elastase (NE), proteinase 3 (PR3), LL37, and four kinds of histones. During NETosis, neutrophils have an ability to engulf pathogens and crystals via producing NETs in both sterile and infectious diseases (22–24).

Although NETs production is beneficial to pathogens clearance, excessive NETs formation may lead to disease conditions in some autoimmune diseases including diabetes (25–27). Previous studies have demonstrated that circulating NETosis markers (MPO, NE) were increased in the serum of DR patients, and high glucose can increase NETs formation *in vivo* and *in vitro* (28, 29). In addition, recent researches proved that NETs have taken part in the pathogenesis of many ocular diseases such as dry eyes and cytokine-induced ocular inflammation (30, 31). However, the specific mechanism that high glucose induces NETs formation in DR patients and the underlying mechanism has not been clearly investigated. It is known that hyperglycemia is the promoter of DR and directly activates other downstream pathways like chronic inflammation condition and oxidative stress (32, 33). Meanwhile, previous studies verified that neutrophils can trigger NETosis in the presence or absence of reactive oxygen species (ROS) (NADPH oxidase-dependent or independent) which is still one of the classical activators NETs formation (24, 34, 35). These evidences may provide a connection between high glucose-induced NETosis and NADPH oxidase pathway.

Herein, we performed this study based on type 2 diabetes mellitus (T2DM) patients and rat models to investigate the ability of neutrophils to undergo NETosis, Then, we investigated the pathways that trigger NETosis in a hyperglycemic environment. We also confirmed the existence of NETs in the retinas of high-fat diet and low dose streptozotocin (STZ)-induced diabetic rats and human vitreous bodies. Finally, the efficiency of anti-VEGF therapy to the deposition of NETs in the eye was taken into consideration.

## Materials and Methods

### Human Blood Samples

This study was approved by the Ethics Committee of Animal and Human Experimentation of Chongqing Medical University. All samples gathering procedures complied with the tenets of the Declaration of Helsinki and ARVO statement on human subjects. All human subjects signed informed consents on their own for participating in this study. Peripheral blood samples were collected from 27 healthy controls (HC) and 90 T2DM patients. Seventy-five T2DM patients were divided into three groups: diabetes without retinopathy (DWR), non-proliferative diabetic retinopathy (NPDR), and proliferative diabetic retinopathy (PDR) (n_DWR_ = 30; n_NPDR_ = 29; n_PDR_ = 31). DM patients were diagnosed according to the diagnostic criteria of the American Diabetes Association (36). The diagnose of DR was determined by well-trained ophthalmologists through fundus photographs based on the revised criteria. Donors with cardiovascular diseases, active infection, autoimmune disease, hematological disease, and high neutrophil count were excluded to avoid potential baseline activation. The control groups were age and gender matched. For *in vitro* experiments, peripheral venous blood was obtained from five healthy donors.

### Human Vitreous Fluid Samples

T2DM patients who had progressed into PDR stage and need vitrectomy except vitreous hemorrhage were involved in this procedure. Vitreous fluid samples (0.1–0.3 mL) were collected from 31 eyes of 30 T2DM patients with PDR before receiving any ocular intervention. The same number of idiopathic macular epiretinal membranes (IMEM) or non-diabetic macular hole (MH) patients were enrolled as normal controls (*n* = 22). To evaluate if anti-VEGF therapy, the classical treatment of DR, have an effect on NETs production, 18 eyes of 18 PDR patients were randomly selected. Only one eye from each patient was included in this study. Vitreous fluid samples were harvested before receiving an intravitreal injection (I.T.I.) with an anti-VEGF drug (Conbercept; 0.5 mg/0.05 mL for each eye. Chengdu Kanghong Biotech Co., Ltd., Sichuan, China) as well as one injection of intravitreal injection. Extracellular DNA/NETs level were measured and compared before and after intravitreal injection.

### Isolation of Serum and Human Primary Neutrophils

Peripheral venous blood was drawn from all donors and gathered in K2-EDTA blood collection tubes or coagulation-promoting vacuum tubes (Becton Dickinson Co.). Serum was obtained through centrifugation (3,000 rpm for 10 min at 4°C) and frozen at −80°C until analysis. Human peripheral blood neutrophils were collected through Ficoll-Dextran methods as described previously (37, 38). After isolation, residual erythrocytes were lysed with red blood cell lysis buffer and washed with PBS. Subsequently, neutrophils were resuspended in normal glucose DMEM medium (5.5 mM, Gibco) which approximates normal blood sugar levels *in vivo* before stimulation. In the end, purified neutrophils (>95%) were assessed by CD16 and CD11b by flow cytometry and counted manually. Cell viability was >95% examined by trypan blue dye.

### Induction of Type 2 Diabetic Rat Model

Type 2 diabetic rat model was induced by high-fat diet and low dose STZ injection. Male Sprague–Dawley rats ranging between 6 and 8 weeks (180–220 g) were purchased from the Animal Care Committee of Chongqing Medical University. All procedures were approved by the Ethics Committee of Chongqing Medical University. Also, they are in line with the Association for Research in Vision and Ophthalmology (ARVO) Statement for the Use of Animals in Ophthalmic and Vision Research. All animals were fed under standard conditions. After adaptive feeding for a week, animals were randomly divided into normal group and diabetic group modeling as previously described (39, 40). The normal group were maintained on their regular diet which consists of 5 fat, 52 carbohydrate, and 20% protein (total kcal value: 20 kJ/kg), while the diabetic group were fed on a high-fat diet consisting of 20 fat, 45 carbohydrate, and 22% protein (total kcal value: 40 kJ/kg) for at least 12 weeks. After 4 weeks, all animals were starved but had free access to water for 12 h before intraperitoneal injection. Rats in diabetic group were treated with a single low dose of STZ (30 mg/kg) dissolved in citrate buffer (pH 4.4), while the normal control group received an injection with a vehicle citrate buffer alone (0.25 ml/kg). After a 1-week injection, we measured non-fasting blood glucose for all rats. Modeling rats whose non-fasting blood levels were >250 mg/dl for three times consecutively were considered to be diabetic, while the others which did not meet these criteria needed a second or third-time injection (30 mg/kg) until they reached these criteria. The controls were given extra injections of citrate as well. After induction of modeling, we detected all animals' weights and blood glucose concentrations each week.

### Isolation and Culture of Primary Bone Marrow-Derived Neutrophils (BMDNs) From Normal and Diabetic Rats

To isolate bone marrow-derived neutrophils, we executed the rats by cervical dislocation and obtained bone marrow cells by flushing all the femur and tibias using normal glucose DMEM medium (5.5 mM). Then we used 52, 64, and 72% Percoll density gradients (GE health, Little Chalfont, UK) to isolate BMDNs as previously described (41). The layer between 72 and 64% Percoll density gradients prefers primary BMDNs.

### Neutrophils Stimulation

For each well in 48 well plates, 5 × 10^6^ Neutrophils were seeded in 200 μl DMEM containing high glucose (5.5, 15, 25, or 35 mM) and PMA (100 nM), and incubated at 37°C with 5% CO_2_ for 120 min. In some experiments, neutrophils were pretreated with diphenyleneiodonium (DPI, 5 μM. MedChemExpress, USA) and Apocynin (10 μM. MedChemExpress, USA), the most widely used NADPH oxidase inhibitors, blocking for 30 min before stimulation.

### Quantification of Extracellular DNA

We used the Quant-iT PicoGreen dsDNA assay kit (Life Technologies, P7589) to measure extracellular DNA to evaluate NETs formation. Pico-Green is a fluorescent dye binding to extracellular dsDNA without staining live cells. Fluorescence signal intensity was measured with a microplate reader (Thermo Fischer) at 485 nm excitation and 535 nm emission. Each sample was measured three times.

### Measurement of Blood-Retinal Barrier Permeability

To measure the function of function and permeability of BRB, animals were deeply anesthetized (10% chloral hydrate, 300 mg/kg) and injected with Evans blue (EB) dye solution through tail vein (45 mg/kg). Then, PBS was used for heart perfusion before the injection of 1% paraformaldehyde (PFA) via the left ventricle after 2 h. The retinas were dissected and dried for weight measuring. After that, each retina was incubated in 200 μl formamide at 70°C overnight and centrifuged for obtaining retinas supernatants. The fluorescence of each sample was measured at 620 and 740 nm (Thermo Fischer).

### Measurement of Retinal Leukostasis

Before intraperitoneal injection, 10% chloral hydrate (300 mg/kg) was used for animal anesthesia in this study. Fluorescein-isothiocyanate (FITC)-coupled Concanavalin A lectin (FITC-CoA; 20 mg/ml in PBS, 5 mg/kg; Vector Labs; Burlington, ON, Canada) was used for label adherent leukocytes and retinal vascular endothelial cells. After deep anesthesia, we opened the chest cavity carefully and inserted the perfusion cannula (14-gauge) into the left heart ventricle. At the beginning of perfusion, rats were perfused with PBS to eliminate erythrocytes and non-adherent leukocytes for over 1 min (500 ml/kg; 35 ml/min). Then we performed a further perfusion with FITC-coupled ConA lectin at 30 ml/min. Later, we separated retinas carefully and observed the morphology of retinal vessels after fixed with 4% PFA. The infiltration of adherent leukocytes in each retina was observed.

### Histological Analysis

After harvesting, all animal eyeballs were fixed with 4% PFA for 24 h. The 10% buffered formalin-fixed retina tissue was embedded in paraffin and sectioned at 5 μm, followed by staining with hematoxylin and eosin (H&E). The paraffin sections were observed and imaged with a fluorescence microscope (Leica, Bannockburn, IL, DM6000).

### Measurement of Reactive Oxygen Species (ROS)

The effect of high glucose on ROS generation was detected by 2′,7′-Dichlorofluorescein Diacetate (DCFH-DA, Sigma-Aldrich, St. Louis, MO, USA) and Dihydroethidium (DHE, BestBio, China). Before stimulation, neutrophils were preloaded with DCFH-DA and DHE (10 μM with 5.5 mM glucose DMEM) for 20 min. Next, we removed the medium and washed the loaded cells with PBS three times. Then, neutrophils were exposed to DMEM medium with different glucose concentrations (5.5, 15, 25, and 35 mM), and stimulation of each concentration lasted for 15 and 30, 60, and 120 min. Finally, the fluorescence signals of DCFH-DA was read at 488 nm excitation and 525 nm emission by the BD FACS Vantage SE Flow Cytometer (BD), and DHE was read at 535 nm excitation and 610 nm emission by a microplate reader (Thermo Fischer).

### ELISA

The concentrations of neutrophil elastase (NE) in the serum of human peripheral blood were assayed with an ELISA kit (JYM, 1078, China) according to the manufacturer's instructions.

### Immunofluorescence and Quantification of NET Formation

For cell staining, primary antibodies against H3Cit (Abcam, ab5103), PR3 (Abcam, ab80705), NE (Abcam, ab68672), and LL37 (Abcam, ab80895) were used to stain the neutrophil-specific enzymes in NETs structure at 4°C overnight and then secondary antibodies were incubated for 2 h at room temperature. As for retinal tissues, we dissected the eyeballs and embedded in OCT (Sakura, USA). Ly6G (BD Pharmingen, 551459) and MPO (Abcam, ab90810) were used as the primary antibodies. DAPI was used for DNA staining. The structure and location of NETs were observed under immunofluorescence microscopy.

### Measurement of Retinal Inflammatory Mediators by Quantitative RT-PCR

All animals were executed to death and the eyeballs were dissected out. The retinal samples were isolated and treated with TRIzol Reagent (Life Technologies, USA). Total mRNA of each sample was extracted according to the manufacturer's protocol. Quantitative PCR was performed using an Applied Biosystems 7500 Fast Real-Time PCR System (Foster City, CA). PCR primers employed were as follows: beta-actin: 5′-CCCTAAGGCCAACCGTGAAAA-3′(forward) and 5′-GGTACGACCAGAGGCATACA-3′(reverse); IL-6: 5′-GCCCTTCAGGAACAGCTATGA-3′(forward) and 5′-TGTCAACAACATCAGTCCCAA GA-3′(reverse); IL-8: 5′-CATTAATATTTAACGATGTGGATGCGT TT CA-3′(forward) and 5′-GCCTACCATCTTTAAACTGCACAAT-3′(reverse); MCP-1: 5′-ATGCAGTTAATGCCCCACTC-3′(forward) and 5′-TTCCTTATTGGGGTCAGCAC-3′(reverse); IL-1β: 5′-CCTTGTGCAAGTGTCTGA AG-3′(forward) and 5′-GGGCTTGGAAGCAATCCTTA-3′(reverse); ICAM-1: 5′-ACGCAGTCCTCGGCTTCTG-3′(forward) and 5′-GGTTCTTGCCCACCTGCTG-3′(reverse); VEGF: 5′-ATCATGCGGATCAAACCTCACC-3′(forward) and 5′-GGTCTGCATTCACATCTGCTATGC-3′(reverse); LY6G: 5′-AACACAACTACCTGCCCC TT-3′(forward) and 5′-CGTTGACAGCATTACCAGTGAT−3′(reverse). Cycling conditions were conducted as described (Polymerase activation at 95°C, 30 s; 40 cycles, 95°C, 5 s; 60°C, 34 s). The data were analyzed using the 2-ΔΔCT method.

### Cell Apoptosis Assay

FITC Annexin V Apoptosis Detection Kit I (556547, BD Pharmingen, USA) were used for apoptosis measurement detected by flow cytometry. 1 × 10^6^ neutrophils in each tube were stimulated by different concentration of glucose and harvested every 30 min up to 120 min as described above, followed by cold PBS wash for twice, and then were resuspended in 100 μl 1 × Binding Buffer. Gently vortex the cells and add 5 μl of FITC Annexin V and 5 μl PI into each tube for 15 min at RT in the dark. Finally, add 400 μl of 1 × Binding Buffer to each tube for analysis by flow cytometry within 1 h.

### Statistical Analysis

All data were normally distributed which were examined by SPSS 22.0 (IBM, USA). All data were expressed as means ± standard error of the mean (SEM) at least for three different replicates of independent experiments. Statistical analysis including paired and unpaired Student's *t*-test, Mann–Whitney test, one-way ANOVA, and Chi-square test was performed using GraphPad Prism version 7.0 software, differences between each group were compared with Tukey's test. Probability values of *P* < 0.05 was statistically significant.

## Results

### Circulating NETosis Products Are Enhanced in the Serum of DR Patients and Diabetic Rats

**Figure 1:**
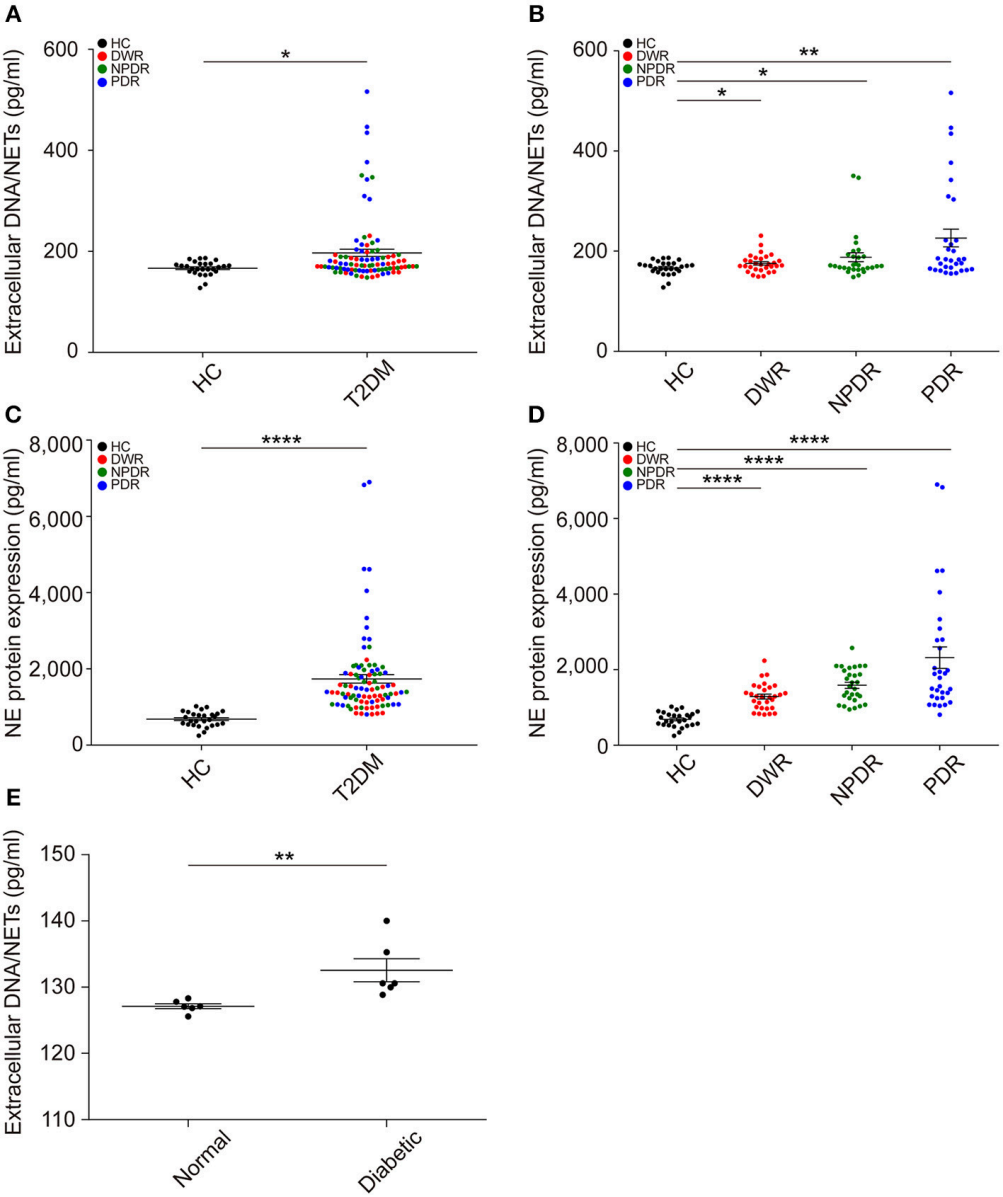
Extracellular NETs and circulating NETosis products are elevated in the serum of diabetic individuals. **(A)** NETs production was measured in the serum of HC and T2DM individuals. *P*-value was assayed by unpaired Student's *t*-test. **(B)** Separated data showing NETs formation in all types of diabetes (DWR, NPDR, and PDR) was obviously increased compared to healthy controls, respectively, which were analyzed by unpaired Student's *t*-test. **(C,D)** Concentration of circulating NE was also examined, which was increased in T2DM patients especially PDR patients compared to healthy controls. n_HC_ = 27; n_DWR_ = 30; n_NPDR_ = 29; n_PDR_ = 31 in above assays. **(E)** Enhanced NETs production in serum of diabetic rats compared to normal controls. n_Normal_ = 6, n_Diabetic_ = 6. Compare to normal rats, *P*-value was assayed by Mann-Whitney test. These assays were repeated for three times. All data were normally distributed. *P < 0.05, **P < 0.01, ****P < 0.0001.

To examine whether long-term hyperglycemia conditions induce NETs formation, we first measured extracellular DNA/NETs and circulating NETosis products NE in the sera of all patients. The results showed that NETs formation was obviously elevated in T2DM patients in the presence or absence of DR (Figure 1A). Then, T2DM patients were divided into DWR, NPDR, and PDR sub-groups. An increased NETs formation was observed in DWR, NPDR, and PDR groups (Figure 1B) when compared with healthy controls. NE expression was also elevated in T2DM patients when compared with healthy controls (Figure 1C). It was also proved that serum NE level was higher in DWR, NPDR, and PDR groups when compared with healthy controls, respectively (Figure 1D). The amounts of increased NETs formation in serum were also observed in high-fat and low dose STZ-induced diabetic rats and normal rats (Figure 1E).

### Hyperglycemia Promotes NETs Formation

**Figure 2:**
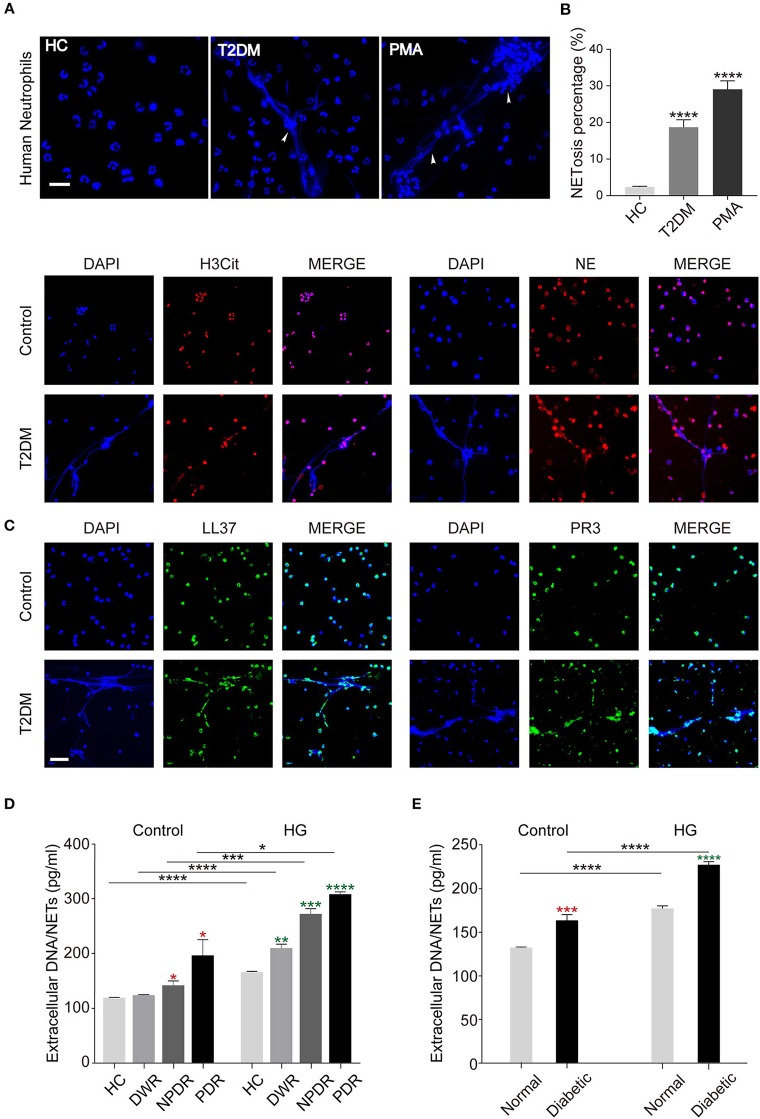
Release of NETs from peripheral blood neutrophils in diabetic individuals. **(A)** Human peripheral neutrophils (PMNs) were isolated from peripheral blood of healthy controls (HC) and T2DM patients and incubated at 37°C for 2 h without any stimulation. PMA served as the positive control. **(B)** Quantification of NETosis percentage of neutrophils. Data were analyzed using Chi-square test. **(C)** Immunofluorescence analysis of high glucose-induced NETs formation determined by co-localization of LL37, PR3, H3Cit, NE, and extracellular DNA. **(D,E)** Quantification of NETs level in PMNs **(D)** and bone marrow-derived neutrophils in animal models **(E)**. The neutrophils were either unstimulated (Control) or stimulated by high glucose (HG, 25 mM). Student's *t*-tests were applied to all the differences indicated by asterisks (red: compared to Control HC or Control Normal; green: compared to HG HC or HG Normal) on error bars. *P < 0.05, **P < 0.01, ***P < 0.001, ****P < 0.0001. Scale bar: 20 μm in **(A)**, and 50 μm in **(C)**.

To explore the effect of hyperglycemia on spontaneous NETosis, neutrophils in peripheral venous blood from T2DM patients with or without DR were determined whether they can form NETs spontaneously. PMA was served as positive control. We examined NETs production with DAPI and found that T2DM patients' neutrophils showed an ability to form NETs spontaneously in the absence of any stimuli, whereas neutrophils from healthy controls hardly produced NETs (Figure 2A). We also did quantification of NETosis percentage of neutrophils and obtained the same results (Figure 2B). To further confirm NETosis, NETs components H3Cit, NE, PR3, LL37, and NET DNA were examined. As shown in Figure 2C, NETs components co-localized to the extracellular chromatin fibers in T2DM patients' neutrophils, rather than healthy controls (Figure 2C), which confirmed the morphology of NETs. Next, we performed a further investigation on whether NETs formation is stimulated by high glucose (HG, 25 mM) *in vitro* in different T2DM subtypes, the result showed that neutrophils from DR patients (NPDR and PDR) can induce more NETs production spontaneously than DWR patients (Figure 2D), additionally, significantly elevated NETs production from PDR patients' neutrophils was observed than the other three groups (Figure 2D). We also isolated BMDNs from diabetic and normal rats followed by HG stimulation, the results were the same as observed in patients (Figure 2E). Thus, these data indicated that hyperglycemic conditions can induce NETs formation by neutrophils. The correlation between diabetic disease course and NETs production has been verified.

**Figure 3:**
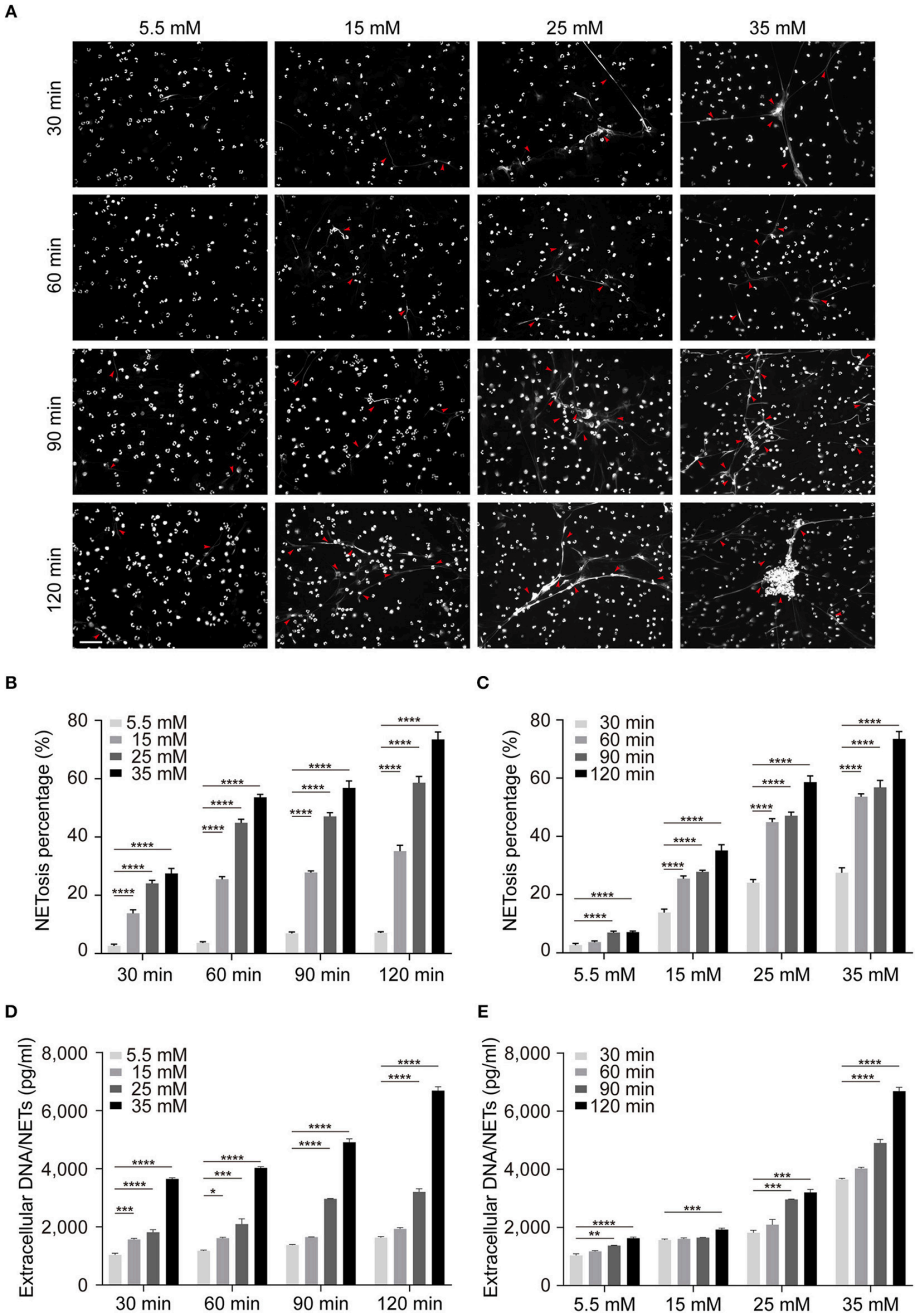
Increased glucose induces more NETs production *in vitro*. The induction of NETosis was conducted on PMNs from healthy controls and treated with different concentrations of glucose (5.5, 15, 25, 35 mM). **(A)** The morphology of NETs (indicated with arrowheads) was observed at different time point (30, 60, 90, 120 min) indicated with DAPI. Scale bar: 50 μm. **(B,C)** Quantification of NETosis percentage of neutrophils. Data were analyzed using Chi-square test. **(D,E)** Release of NETs was quantified from every 30 min up to 120 min. Differences were accessed by one-way ANOVA. *P < 0.05, **P < 0.01, ***P < 0.001, ****P < 0.0001.

In order to further illustrate the influence of glucose on NETosis, we determined the impact of glucose on NETs production in normal cells *in vitro*. We isolated neutrophils from three healthy individuals and incubated them in DMEM with different glucose concentration (5.5, 15, 25, and 35 mM) and different time point (30, 60, 90, and 120 min) to determine the correlation between NETs formation and stimulation conditions. Mannitol was set as hypertonic control and showed no ability in NETosis induction. During the 2 h incubation under high glucose conditions, most nuclear decondensed and netting neutrophils formed with time prolonging (Figure 3A). The results based on both microscopic images and fluorometric analysis showed that increased NETosis percentage (Figures 3B,C) and NETs level (Figures 3D,E) depended on exposure time to and concentration of glucose, which were not caused by apoptosis (Figure S1 in Supplementary Material).

### High Glucose Induces NETs Formation Through an NADPH Oxidase-Dependent Pathway

**Figure 4:**
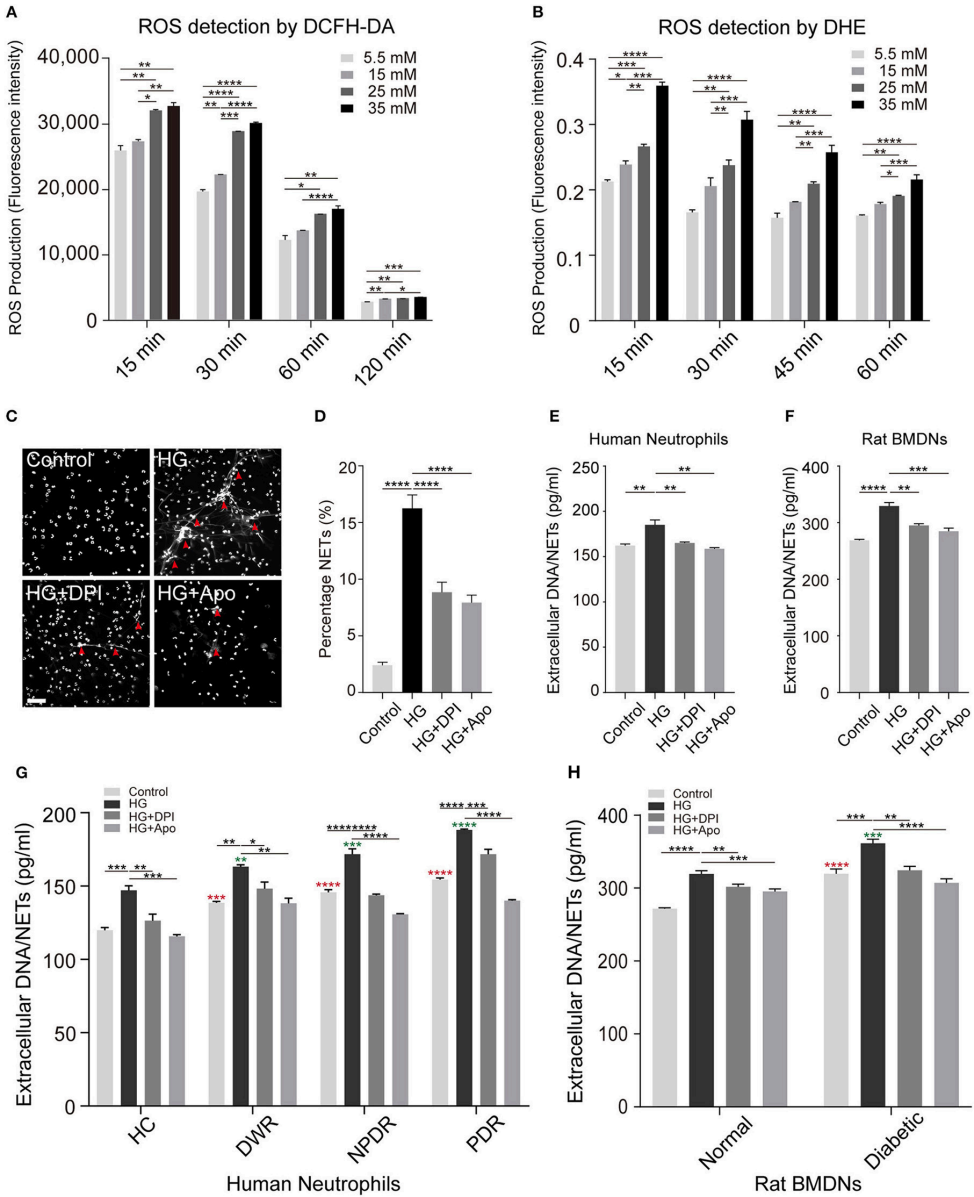
Increased concentrations of glucose promotes ROS production and NETs formation. **(A)** Glucose-induced ROS production quantified through flow cytometry preload with DCFH-DA probe dyes. **(B)** Glucose-induced ROS production measured with a microplate reader preload with DHE probe dyes. One-way ANOVA with Tukey's test were used to access the differences. **(C)** Neutrophils were pretreated with diphenyleneiodonium (DPI, 5 μM; Apocynin, 10 μM) for 30 min followed by any stimulation for 120 min. The morphology of NETs (indicated with arrowheads) was observed through fluorescence microscopy. Scale bar: 50 μm. **(D)** Quantification of NETosis percentage of neutrophils. Data were analyzed using Chi-square test. **(E,F)** Neutrophils from healthy human controls (*n* = 5) and BMDNs from normal rats (*n* = 3–4) were treated with different kinds of stimuli for 2 h. Differences between HG and other groups were compared by one-way ANOVA. **(G,H)** Measurement of NETs level of neutrophils from both human individuals and rats received different stimulus (n_HC_, n_DWR_, n_NPDR_, n_PDR_ = 3–4; n_Normal_ = 6, n_Diabetic_ = 6). One-way ANOVA was used to analyze the differences indicating by asterisks (red: compared to Control HC or Control Normal; green: compared to HG HC or HG Normal) on error bars. *P < 0.05, **P < 0.01, ***P < 0.001, ****P < 0.0001.

To assess whether high glucose-induced NETs formation is ROS-dependent, neutrophils were exposed to DMEM with different glucose concentration preloaded with DCFH-DA or DHE. Upon HG stimulation (25 and 35 mM), neutrophils displayed enhanced ROS levels (Figures 4A,B). Our data supported the fact that the dynamic changes in the rate of NETs production are associated with glucose-induced overproduction of ROS. Time-course experiments showed that ROS production declined after 15 min (Figures 4A,B). The results verified significant neutrophil oxidative burst during high glucose stimulation (Figures 4A,B). For further illustration whether ROS has an effect on NETs modulation, DPI and Apocynin, two inhibitors of NADPH oxidase, were used to pretreat neutrophils before high glucose stimulation. In these conditions, neutrophils displayed less potential to form NETs (Figures 4C,D). Additionally, NETs formation was analyzed by quantifying extracellular DNA in the cell supernatants in both healthy human neutrophils and BMDNs from normal rats after stimulation or inhibition. Inhibition of NADPH oxidase significantly reduced the formation of extracellular DNA when compared to HG stimulation in both normal human and rat neutrophils (Figures 4E,F). Moreover, same results were observed in both T2DM patients and diabetic rats (Figures 4G,H). All these procedures affirmed that high glucose-induced NETs formation is NADPH oxidase-dependent.

### Neutrophils Infiltration Cause Retinal Leukostasis and Chronic Low-Grade Inflammation

**Figure 5:**
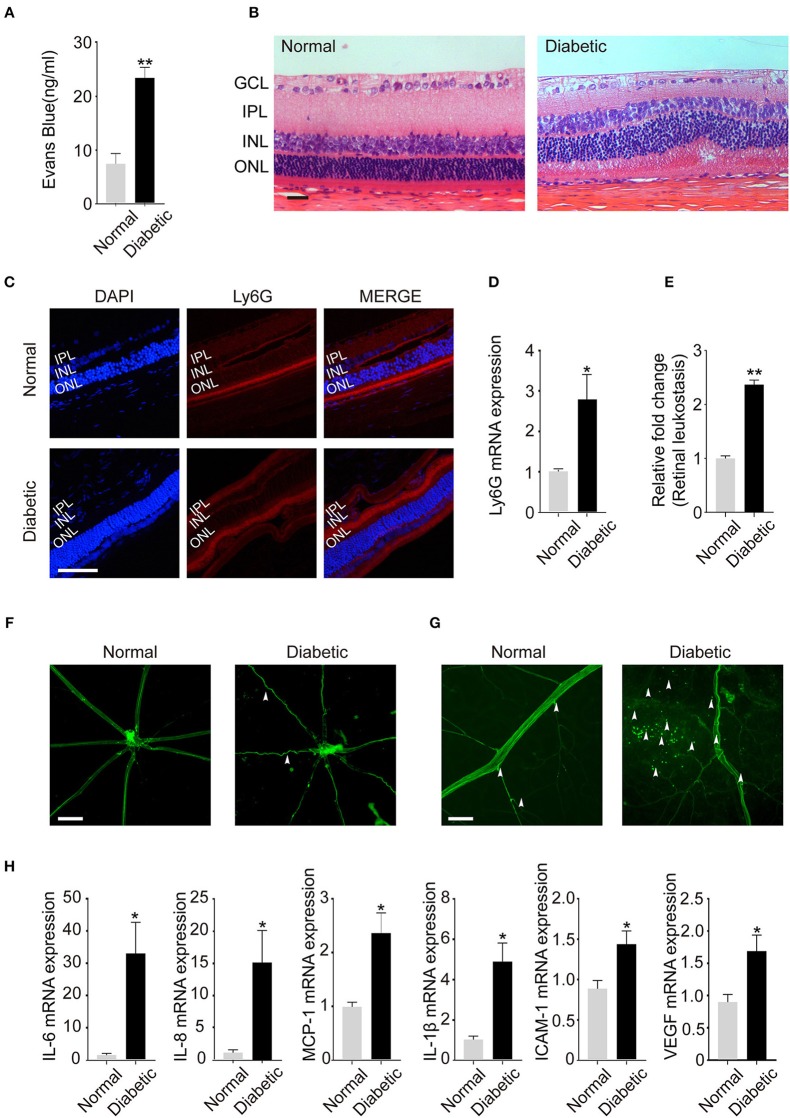
Neutrophil Infiltration in the retina lead to retinal leukostasis and cause chronic low-grade inflammation in the retinas of rat models. **(A)** The permeability of the blood–retinal barrier (BRB) was examined with the Evans blue (EB) permeation experiment (n_Normal_ = 3, n_Diabetic_ = 3). **(B)** Representative pathology photomicrographs of H&E staining of retinopathy (GCL: ganglion cell layer, IPL: inner plexiform layer, INL: inner nuclear, ONL: outer nuclear layer). **(C)** Immunofluorescence analysis of neutrophil marker Ly6G to label the location of neutrophils of retinas. (INL: inner nuclear, ONL: outer nuclear layer). **(D)** Expression of neutrophil marker Ly6G **(C)** in the retinas quantified by qRT-PCR. **(E)** Quantification of retinal leukostasis. Data were analyzed using Mann–Whitney test. (n_Normal_ = 5, n_Diabetic_ = 5). **(F)** FITC-coupled ConA lectin revealed the shape of retinal vasculature (indicated with arrowheads); **(G)** Adherent leukocytes and fluorescein leakage in retinal vessels are indicated with arrowheads. **(H)** The expression of IL-6, IL-8, MCP-1, IL-1β, ICAM-1, and VEGF in the retinas was quantified by qRT-PCR. Data were analyzed by Mann-Whitney test. *P < 0.05, **P < 0.01. Scale bar: 20 μm in **(B,C)**, 100 μm in **(F)**, and 50 μm in **(G)**.

To further investigate whether NETosis was associated with DR, we verified NETs deposition in type 2 diabetic rat models. The body weights of diabetic rats were higher than normal controls after 4-week feeding. Random blood glucose concentration of diabetic rats became stable after successful modeling. Meanwhile, they showed the characteristics of diabetes including polyuria, polydipsia, and polyphagia. After 3 months of induction, which refers to the stage of retinopathy, we used Evans-Blue (EB) technique to measure the permeability and function of the retinas in both groups. The results showed that BRB function broke down and increased EB extravasation was observed in diabetic group compared to normal control (Figure 5A). H&E staining revealed retinal structure disorganization in diabetic rats, both the inner and outer layer structures were disordered (Figure 5B). Thus, all these findings suggested that BRB broke down in diabetic rats, which provide a precondition for following studies.

As a chronic low-grade inflammatory disease, DR is characterized by the activation of inflammatory cells especially neutrophils. Previous studies have proved that neutrophils undergo NETosis in hyperglycemic conditions after releasing into peripheral blood. Therefore, we examined the existence of neutrophils in the eyes of diabetic objects, and we first used Ly6G, the specific neutrophil marker, to determine the location of neutrophils in retinas. Neutrophils were observed in the vitreous bodies and retinal structures especially the inner nuclear layer (INL) and outer nuclear layer (ONL) in both diabetic and normal rats (Figure 5C). Meanwhile, Ly6G mRNA level was significantly increased in the retinas of diabetic rats (Figure 5D), which confirms neutrophils presence in the eyes.

It is acknowledged that neutrophils are the first defenders at the sites of infections and are pivotal in NETs induction. Moreover, inflammatory cells infiltration and adhesion to vascular endothelial cells are the specific characteristics of DR. Therefore, FITC-coupled ConA lectin was used to demonstrate the adherent leukocytes and retinal vasculature of the retinas. We observed that the shape of retinal vasculature became tortuous and dilated when DR happened (Figure 5E). In addition, there were obvious fluorescein leakage and elevated adherent leukocytes in retinal vessels in the diabetic group (Figures 5E–G).

Besides, chronic low-grade inflammation, which is also correlated with the onset of DR, may be caused by inflammatory cells like neutrophils. In this condition, we examined the expression of the inflammatory, adhesion, and angiogenic factors mediators such as IL-6, IL-8, MCP-1, IL-1β, ICAM-1, and VEGF in the retinas. All cytokines were significantly increased in the retinas of diabetic rats (Figure 5H), which confirmed chronic low-grade inflammation in the retinas of diabetic models. These data indicated that increased neutrophil infiltration in the retinas may accelerate neutrophil-endothelial cell adhesion, retinal leukostasis, and inflammatory reactions, which contributes to the disease progression.

### NETs Deposit in the Vitreous Body and Retina and VEGF May Involve in the High Glucose-Regulated NETosis

**Figure 6:**
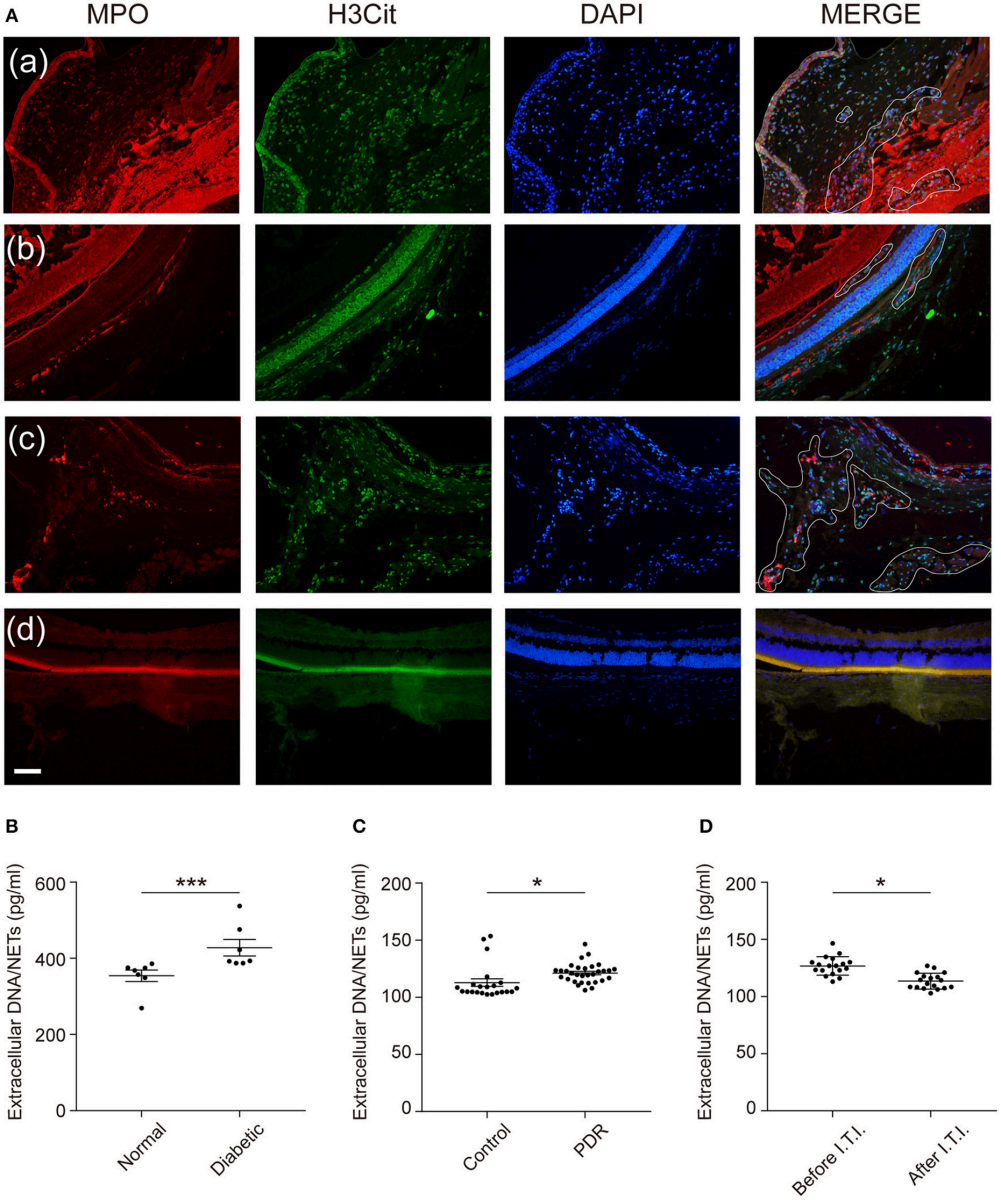
NETs deposit in the vitreous bodies of DR patients as well as retinas of diabetic rats, and VEGF downregulate high glucose-induced NETosis. **(A)** Representative immunofluorescence images for co-localization of NETs components H3Cit, MPO, and DAPI in the retinas (defined by white line). Scale bar: 20 μm (a–c: diabetic rats; a: anterior chamber; b,c: posterior chamber; d: normal rats, posterior chamber). **(B)** NETs were quantified in the retinas of rats. Data were analyzed by Mann-Whitney test. **(C)** NETs were quantified in vitreous fluid in human subjects (n_Control_ = 31, n_PDR_ = 22). Differences were accessed by unpaired Student's *t*-tests. **(D)** NETs were measured in vitreous fluid in PDR patients after receiving an intravitreal injection (I.T.I.) and before I.T.I. treatment (n_BeforeI.T.I._ = 31, n_AfterI.T.I__._ = 22). Paired Student's *t*-test was performed to evaluate the differences. *P < 0.05, ***P < 0.001.

Next, we aimed to demonstrate whether infiltrated neutrophils took part in the formation of NETs in the eyes. We labeled typical NETs with markers H3Cit and MPO. As expected, the typical structures of NETs were found in the vitreous bodies and retinas of diabetic rats, which were presented as decondensed DNA structures and co-localized proteins (Figure 6A). Due to the existence of NETs structure in the retinas, we analyzed extracellular NETs/DNA in the retinas in the following study. The results showed a significantly higher level of extracellular NETs/DNA in diabetic rat retinas than control groups (Figure 6B), which confirmed NETs existence in retinas of DR individuals. We also verified extracellular DNA/NETs level was higher in vitreous fluid in PDR patients compared to the normal controls (Figure 6C). To test whether anti-VEGF therapy, the classical treatment of DR, has an effect on NETs production, we selected 18 eyes of 18 PDR patients and measured Extracellular DNA/NETs level before and after receiving an intravitreal injection (I.T.I.) of the anti-VEGF drug. A significant decrement of NETs production was observed after anti-VEGF therapy (Figure 6D).

## Discussion

Previous studies have found that circulating NETs components products such as NE, MPO, and extracellular DNA/NETs are increased in the serum of DR patients (29). However, the underlying mechanism about NETs formation in the neutrophils of DR patients and whether NETs formation takes part in the pathogenesis of DR has not been clearly investigated. Our study was the first to demonstrate that NETs present in the serum as well as eye tissues of diabetic individuals and are promoted spontaneously by high glucose via an NADPH oxidase-dependent way.

Our data agree with previous studies which showed NETs could be induced by classical inducer PMA and high glucose (27, 28). In addition, we also proved that NETs are elevated in the serum of diabetic individuals, which is consistent with previous studies (27, 28). It has been reported that long-term hyperglycemia is associated with the degree of diabetic complications (42). Thus, we divided diabetic patients into different stages based on the degree of retinal pathology and found that NETs formation was much higher in PDR patients, the stage which is characterized by BRB breakdown and neovascularization, compared to DWR patients. This may suggest a correlation between disease course and NETs production.

Based on the serological results mentioned above, we pursued further studies to verify the ability of neutrophils forming NETs. In this study, we observed that neutrophils from diabetic individuals could promote spontaneous NETosis compared with healthy controls. Moreover, neutrophils from healthy individuals could produce NETs stimulated by high glucose. Our observations ensured the influence of hyperglycemia and high glucose on NETs production.

Since multiple types of research proved that NETosis can be triggered through NADPH oxidative-dependent or independent pathway under different stimuli, meanwhile, the activation of oxidative stress by neutrophils is a critical pathway in DR. Therefore, the influence of glucose on cellular ROS production in human neutrophils needs to be investigated. Our data showed that ROS level was significantly higher in the process of high glucose-induced NETosis, which verified the involvement of NADPH oxidase activation. In addition, DPI successfully inhibited high glucose-induced NETs formation, which verified that NETs formation is ROS-dependent. Thus, we found that high glucose-modulated NETosis is NADPH oxidase-dependent. These may suggest a new therapy target in diabetic treatment.

Next, in order to evaluate whether NETs formation is enrolled in the pathogenesis of DR, we verified neutrophils exist in the eyes based on well-established animal models. When DR happens, neutrophils are released from peripheral blood, then infiltrate and adhere to vascular endothelial cells on account of BRB breakdown, which are observed in our study. Moreover, once we confirmed the presence of neutrophils in the eye, we needed to explore whether NETs deposit in the eye in further steps, which may provide the association between the pathogenesis of DR and NETs formation. We afforded strong proofs that NETs present in both retinas of diabetic rats as well as DR patients' vitreous bodies. Our data proved that the observation of NETs in the eye is caused by peripheral neutrophils when BRB breakdown.

Furthermore, we evaluated whether anti-VEGF therapy is valid in NETs reduction. Decreased NETs formation was found in PDR patients' vitreous fluid after I.T.I. therapy, which may manifest that VEGFR2-mediated pathway may involve in the downregulation of NETs formation. It has been reported that ROS promotes the production of VEGF, and VEGF promotes ROS production derived from NADPH oxidase in turn. Besides, ROS are critical chemical species in VEGFR2-mediated signaling, which lead to neovascularization in DR (42, 43). It has been reported that anti-VEGF drug functioned by combining with specific receptors (VEGFR) in the vascular endothelial cells in DR patients (43). Thus, our findings indicate that NADPH oxidase-derived ROS may be another signaling pathway involved in anti-VEGF therapy.

We also found the serological results of NETs quantification agree with the intravitreal results, which may indicate that higher level of NETs may involve in the pathogenesis of DR. Although the NPDR and DWR vitreous samples cannot be collected, we still observed the elevation of NETs in PDR patients. This may provide a way for evaluating the degree of DR by monitoring NETs circulating products in the serum of diabetic patients to prevent disease from progressing and give timely treatment.

In conclusion, our data illustrated that NETosis can be triggered by neutrophils under high glucose circumstances. The specific mechanism of how high glucose-induced NETs formation was found to be related to oxidative stress machinery. Long-termed hyperglycemia provides peripheral neutrophils with more opportunities to cross the BRB, form NETs and deposit in the vitreous body as well as the retina. Meanwhile, anti-VEGF therapy can reduce NETs formation in the vitreous body, which may suggest a new target for therapy in DR. Our study didn't explore the specific mechanism by inhibition of ROS and VEGF in rat models. Further studies need to evaluate the efficiency of new target treatment through DR disease models by intravitreal injection.

## Author Contributions

LW and XuZ designed the research project. LW conducted the experiments and interpreted the data. LW and XZ prepared the figures, wrote the manuscript. YM contributed to animal modeling and raising. YY and DW collected all the clinical samples. LW, XZ, and XuZ revised the manuscript. All authors reviewed the manuscript.

### Conflict of Interest Statement

The authors declare that the research was conducted in the absence of any commercial or financial relationships that could be construed as a potential conflict of interest.
