# Supplementary material for: Hyperglycemia Induces Neutrophil Extracellular Traps Formation Through an NADPH Oxidase-Dependent Pathway in Diabetic Retinopathy
Source: Front Immunol. 2019 Jan 8;9:3076. doi: 10.3389/fimmu.2018.03076 (PMC6331470; doi:10.3389/fimmu.2018.03076)
Supplement: Supplementary file 1 [file Data_Sheet_1.PDF]

Supplementary Material

Hyperglycemia Induces Neutrophil Extracellular Traps Formation through an NADPH Oxidase-Dependent Pathway in Diabetic Retinopathy

Luoziyi Wang*, Xin Zhou, Yizhou Yin, Yuxin Mai, Desai Wang, Xuedong Zhang*

*** Correspondence:** Xuedong Zhang: zxued@sina.com

Xin Zhou, Yizhou Yin, Yuxin Mai, Desai Wang, Xuedong Zhang*

1.1 Supplementary Figures

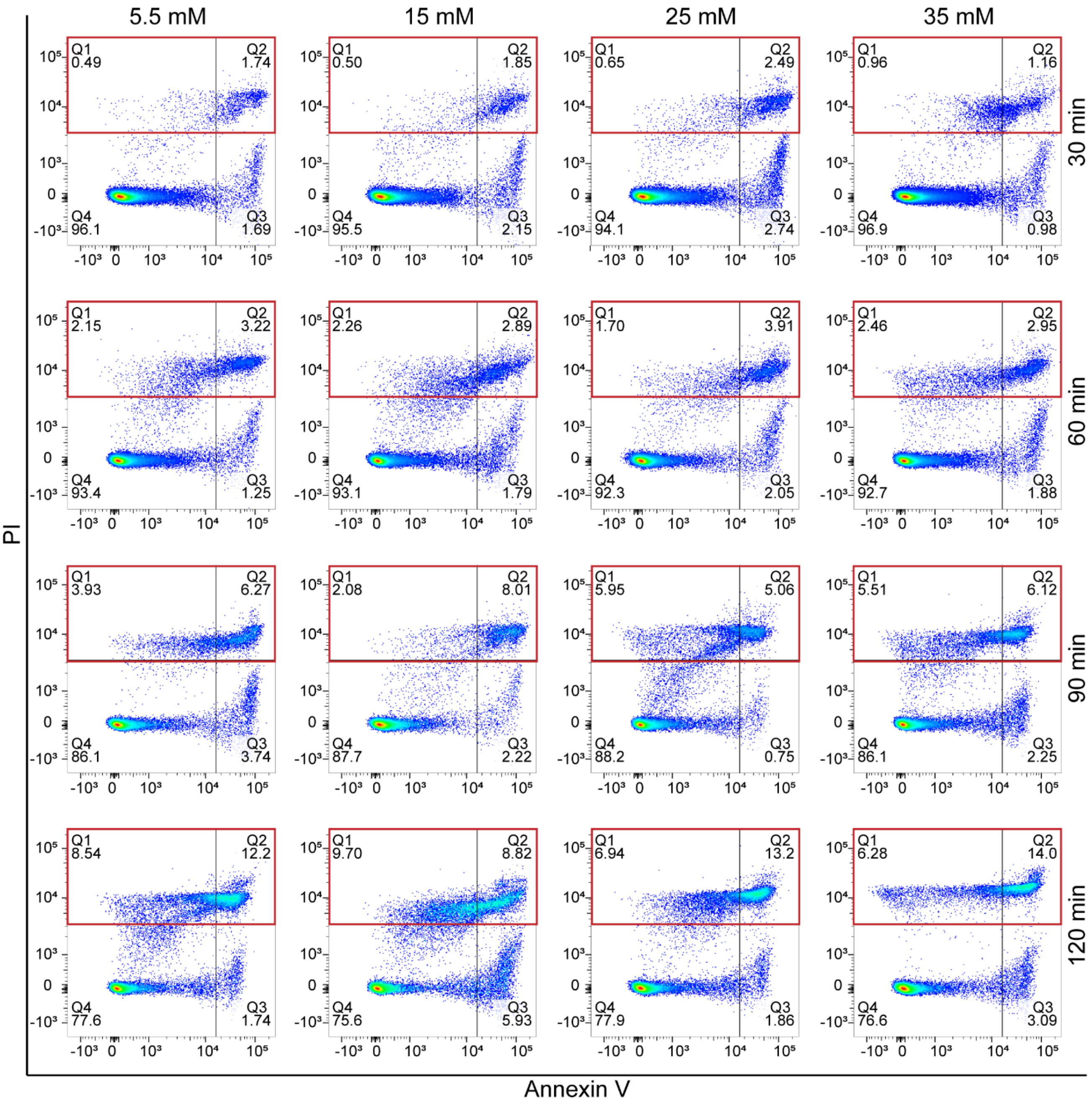

Supplementary Figure 1. | Apoptosis of neutrophils after stimulation with HG. Annexin V and PI were used for measuring apoptosis rate of neutrophils by flow cytometry. Cells in red boxes were PI positive, which may disrupt the examination of extracellular DNA/NETs level, showed no significant difference between different concentration of glucose at the same time point.
